# Transitioning to Digital Systems: The Role of World Health Organization’s Digital Adaptation Kits in Operationalizing Recommendations and Interoperability Standards

**DOI:** 10.9745/GHSP-D-21-00320

**Published:** 2022-02-28

**Authors:** Tigest Tamrat, Natschja Ratanaprayul, Maria Barreix, Özge Tunçalp, David Lowrance, Jenny Thompson, Leona Rosenblum, Nancy Kidula, Ram Chahar, Mary E. Gaffield, Mario Festin, James Kiarie, Brian Taliesin, Carl Leitner, Sylvia Wong, Teodora Wi, Hillary Kipruto, Ayotunde Adegboyega, Derrick Muneene, Lale Say, Garrett Mehl

**Affiliations:** a UNDP/UNFPA/UNICEF/WHO/World Bank Special Programme of Research, Development and Research Training in Human Reproduction (HRP), Department of Sexual and Reproductive Health and Research, World Health Organization, Geneva, Switzerland.; b World Health Organization, Department of Digital Health and Innovations, Geneva, Switzerland.; c World Health Organization, Department of Global HIV, Hepatitis and Sexually Transmitted Infections Programmes, Geneva, Switzerland.; d PATH, Digital Square, Seattle, WA, USA.; e John Snow Inc., Center for Digital Health, Washington. DC, USA.; f World Health Organization Regional Office for Africa, Multicountry Assistance Team, Kampala, Uganda.; g World Health Organization Country Office for India, Maternal & Reproductive Health Team, New Delhi, India.; h University of Philippines, College of Medicine, Manila, Philippines.; i PATH, Living Labs, Nairobi, Kenya.; j PATH, Digital Square, Chapel Hill, NC, USA.; k United Nations Population Fund, New York, NY, USA.

## Abstract

**Introduction:**

The transition from paper to digital systems requires quality assurance of the underlying content and application of data standards for interoperability. The World Health Organization (WHO) developed digital adaptation kits (DAKs) as an operational and software-neutral mechanism to translate WHO guidelines into a standardized format that can be more easily incorporated into digital systems.

**Methods:**

WHO convened health program area and digital leads, reviewed existing approaches for requirements gathering, mapped to established standards, and incorporated research findings to define DAK components.

**Results:**

For each health domain area, the DAKs distill WHO guidelines to specify the health interventions, personas, user scenarios, business process workflows, core data elements mapped to terminology codes, decision-support logic, program indicators, and functional and nonfunctional requirements.

**Discussion:**

DAKs aim to catalyze quality of care and facilitate data use and interoperability as part of WHO’s vision of SMART (Standards-based, Machine-readable, Adaptive, Requirements-based, and Testable) guidelines. Efforts will be needed to strengthen a collaborative approach for the uptake of DAKs within the local digital ecosystem and national health policies.

## Abstract (precis)

The World Health Organization (WHO) digital adaptation kits distill WHO guidance into a standardized format that can be more easily incorporated into digital systems and facilitate communication between the health workforce and technologists to enable a shared understanding of the underlying content.

## Key Messages

- Digital adaptation kits (DAKs) translate World Health Organization (WHO) guidelines for a health domain area into a package of business process workflows, core data needs, decision-support algorithms, linkages to indicators, and functional requirements that can then be more easily translated into digital systems.
- DAKs are part of the suite of tools within the WHO SMART (Standards-based, Machine-readable, Adaptive, Requirements-based, and Testable) guidelines approach to systematically reinforce clinical, public health, and data recommendations within digital systems.
- DAKs serve as a critical step in fulfilling WHO’s long-term vision of SMART guidelines for transforming guideline development, delivery, and application in the digital age. A key measure of success will be ensuring countries can adapt the generic DAKs according to their digital ecosystem and aligned to their national health policies.
- Collaborations across health program leads, digital health and health information systems focal points, implementers, software developers, and service providers will be critical for the effective use of DAKs within country contexts.

## INTRODUCTION

Countries are increasing investments into the digitization of legacy paper-based systems, such as registers and tally sheets, as part of efforts to improve quality of care, data use, and accountability, as well as to reduce the clerical burden on health workers.^1^^–^^5^ This introduction of digital tools may be done through electronic medical records or systems to track an individual’s (e.g., pregnant woman or child) health status and services received.^5^^,^^6^ Despite the various entry points, the digitization process provides an opportunity not only to automate paper-based forms into electronic formats but also to capitalize on the added benefits of digital tools, such as improving care through embedding decision-support derived from evidence-based recommendations. For countries to effectively benefit from these digital investments, systems need to be designed appropriately to ensure accuracy of the health content (e.g., decision-support logic), streamline data needs, and enforce the use of data standards for interoperability. We present a systematic approach developed by the World Health Organization (WHO) for point-of-care digital systems to reinforce service delivery protocols and capture individual-level data to fulfill the needs of health information systems.

Although quality assurance of the clinical and data content within digital systems (also known as technical requirements) is critical for maximizing the health impact of digital tools, there are several challenges in documenting and reviewing the specifications that guide the design of digital systems. One common challenge is the interpretive process required in distilling the narrative text in paper-based resources and defining the clear inputs needed for a digital system.^7^^–^^11^ Evidence-based guidelines, such as those published by WHO, offer a validated starting point for defining decision-support requirements.^12^ However, these recommendations, available as narrative text, often do not provide sufficient detail to be converted into the technical specifications that software developers require.^7^^–^^9^^,^^11^ This, in turn, can lead to potential misinterpretation, inability to trace how decisions were defined, or omit the recommendations altogether due to the effort required to translate them for the digital system.

Evidence-based recommendations, available as narrative text, often do not provide sufficient detail for software developers to convert into the technical specifications they require.

Determining the appropriate data elements and embedding terminology standards is another consideration in transitioning from paper to digital systems. Data collection in legacy, paper-based systems often requires redundant tabulation that is burdensome for health workers and requires significant levels of consolidation before they can be incorporated into digital systems.^13^ Digital systems offer the opportunity to capture data once and harness it for multiple needs, such as prompting decision-support logic, surveillance, and reporting; however, this requires meticulous planning to streamline across various paper-based tools (e.g., registers) and data collection processes.^13^^,^^14^ Furthermore, as paper-based systems become digitized, there is also a need to embed interoperability standards to support continuity of care and data exchange, which may often be deprioritized during software development.

Beyond facilitating quality assurance, transparent documentation of technical requirements is important to mitigate dependence on specific software development companies (e.g., vendor lock-in) and increase efficiencies for scaling digital systems. However, this documentation is often unavailable or may be proprietary, requiring governments and technology partners to start from scratch and expend significant resources each time they intend to build and adapt such systems. Previous efforts to develop accessible requirements for informing the content of digital systems provide a solid foundation, but they lack operational components, such as data dictionaries and decision-support logic.^15^^–^^18^ In instances where data dictionaries and decision-support logic are publicly available, they are tied to specific software applications,^19^^,^^20^ may not apply data standards, or have not been generalized for use across different contexts.^21^^,^^22^ Furthermore, requirements gathering activities, which define the content and functionalities of digital systems, are primarily targeted at the technology teams, rather than the health program leads who are responsible for verifying the information within these systems.

Based on these findings, WHO developed digital adaptation kits (DAKs) as an operational and software-neutral mechanism that distills WHO recommendations into a standardized format that can be more easily incorporated into digital systems. The DAKs are intended to provide comprehensive technical requirements to apply WHO guidance in digital form. They also aim to facilitate communication among health practitioners/managers and technologists as they design digital systems to enable a common understanding of the underlying content within digital systems.

DAKs are part of a suite of tools within a broader WHO-driven effort known as SMART— Standards-based, Machine-readable, Adaptive, Requirements-based, and Testable—guidelines to improve the fidelity and uptake of recommendations within standard-based digital systems.^12^ The SMART guidelines framework outlines an incremental 5-level pathway for translating narrative guidelines for digital systems through derivative products, such as machine-readable recommendations that can be encoded into digital systems and reference software applications.^12^ DAKs represent the first step within the SMART guidelines vision in navigating this transition. We describe the methods and key findings in developing DAKs for sexual and reproductive health (SRH) and HIV, the first set of health program areas within WHO to use this approach. This article describes a replicable model for health programs and outlines lessons learned and key next steps in this evolving process.

We describe the methods and key findings in developing DAKs for SRH and HIV, the first set of health program areas within WHO to use this approach.

## METHODS

Three fundamental principles guided the methodology for establishing the DAKs.

1. Derive the technical requirements from WHO clinical, public health, and data use guidance and maintain a generic framing that can be localized across different settings. A rule of thumb of 80% generic content and 20% requiring local contextualization served as an underlying assumption. For example, approximately 80% of the WHO guideline recommendations will apply to most countries, but 20% may require adaptation to the local context such as tailoring which health services specific cadres can provide to clients.
2. Create the resulting documentation to be software neutral, meaning that it had to be independent of a branded software system and can be used to develop and update any digital system that supports functionalities for tracking health services and decision support.
3. Format the components within the DAKs to be understandable by both clinical and data managers and those involved in the development of digital systems, serving as a common language for communication among these diverse stakeholder groups.

### Determining the DAK Components

To define components within the DAKs, WHO worked with colleagues across the headquarters, regional, and country level, as well as with external collaborators with expertise in requirements gathering and/or SRH domains. This team used an iterative process that combined desk reviews of requirements gathering practices, findings from digital implementations, review of existing paper-based tools, and stakeholder consultations.

The Collaborative Requirements Development Methodology, an established approach in requirements gathering^15^^–^^17^^,^^23^ guided the structure for the DAKs and served as the basis for creating the following DAK components: personas, business process workflows, and functional and nonfunctional requirements. Experiences from a WHO multisite study on digitizing paper-based systems complemented the gaps observed in the Collaborative Requirements Development Methodology approach. For example, the multisite study demonstrated the need to provide data dictionaries and decision-support logic, such as algorithms and service delivery schedules, in a modifiable spreadsheet format.^24^ The findings from the WHO multisite study also helped to define the structure of the data dictionary by specifying the required data types for presenting the data dictionary in a usable format for software developers to execute in digital systems. This data structure was also aligned with the requirements for compliance with Health Level Seven International Fast Healthcare Interoperability and mapped to the International Classification of Diseases (ICD) 11 concepts.

Experiences from a WHO multisite study on digitizing paper-based systems complemented the gaps observed in approaches used to structure the DAKs.

Lastly, a series of stakeholder consultations with individuals representing clinical, digital, and health informatics expertise and spanning country, regional, and global perspectives were held periodically between August 2018 and July 2019 to refine the structure of the DAKs and finalize the main components.

Once these components were identified, the methodology team, comprising of WHO staff (NR, TT, and GM) and consultants supporting this work (CL, BT, and JT) leveraged existing documentation and notation standards, such as business process modeling notation for the workflows,^25^ XLSForm for the data dictionary, and decision model and notation for elaborating the decision logic.^26^ External stakeholders also reviewed and validated the structure of these components. Slight adaptations were made in using these informatics notations to make the format accessible to a health audience with minimum technology background but still retained the consistency needed for informaticians and technology developers. For example, the DAKs applied a simplified version of Business Process Model and Notation that used only a subset of symbols that were common and available across various workflow mapping software tools, as opposed to using the full range of notations. The decision-support tables, based on the Decision Model and Notation standard, included additional rows to provide greater narrative, workflow triggers, and annotations on what could be included in the prompts provided to the health worker.

### Populating the DAK Components

For each health area of the DAKs, the methodology team and WHO SRH/HIV domain leads (MB, ÖT, MF, MLG, DL, JK, and TW) worked together to populate the details of each DAK component.

For personas, the roles and responsibilities were based on global WHO guidance on the official classification of health worker roles^27^ and task-shifting recommendations for reproductive, maternal, newborn, and child health services,^28^ with the assumption that local implementations can be contextualized through user-centered design principles.^29^

Workflows were created based on a review of existing documentation, where possible, and a series of workshops that included role-play to help illustrate the progression of activities.^15^ In the case of family planning (FP), implementing partner organizations also conducted observations with health workers during their routine activities across various countries to contextualize and validate the workflows. All workflows were further reviewed with clinical experts at the country and regional levels to confirm their relevance.

Then, user scenarios were developed through storytelling and role-plays to narrate the common activities that take place during a service delivery encounter.

Data elements for the data dictionary were derived in an iterative manner working backward from inputs for the decision-support logic, indicator calculations, and recommendations from primary clinical data collection tools.^30^ Service delivery registers from different settings were reviewed to inform the development of the data dictionary and ensure the process was grounded in the realities of routine clinical settings. A substantial amount of effort was required to standardize and detail the data element definition, data type (e.g., multiple choice or string), response options, skip logics, calculations, and other requirements for coherence across data elements. Once the data dictionary was completed, data elements were mapped to terminology standards, including the ICD.

The algorithms for the decision-support logic were developed primarily through the triangulation of relevant guidelines and feedback from clinical leads involved in the guideline development. As guideline content is not readily transferable for execution within digital systems, this required a series of consultations with the technical lead of the WHO guidelines and clinical experts to clarify the algorithms for the decision-support logic. For antenatal care, a clinical expert deconstructed the inputs and outputs in terms of decision trees and “if/then” statements for each of the clinical recommendations included in the 2016 WHO guideline recommendations on antenatal care for a positive pregnancy experience.^31^^,^^32^ In the case of FP, algorithms developed for the medical eligibility criteria wheel application^33^ were repurposed and adapted to align with the DAK template and notation standards.

The indicators component was based on existing WHO guidelines on recommended aggregate indicators for health management information systems-based monitoring of the health program areas.^34^^,^^35^ These resources typically included the numerators and denominators required for computing the indicators, which were then linked to the core data elements (individual-level data) in the data dictionary. Similarly, the list of functional requirements was based on existing resources^15^^,^^16^ and discussions with the stakeholders.

## RESULTS

The combination of the Collaborative Requirements Development Methodology approach, multisite research findings, and stakeholder consultations established the 8 components of the DAKs. For each DAK health domain, the components consist of (1) health interventions and associated recommendations (e.g., maternal and fetal assessment during pregnancy); (2) generic personas; (3) user scenarios; (4) generic business processes and workflows; (5) core data elements; (6) decision-support logic; (7) indicators and performance metrics; and (8) functional and nonfunctional requirements (Table 1). Outputs, such as spreadsheets of data dictionaries and detailed decision-support algorithms, are appended as part of the DAKs. Examples of these different components from SRH and HIV are available in the Supplement. All components were validated during the stakeholder consultations.

**TABLE.** Components of the World Health Organization Digital Adaptation Kit

**Component and Description**	**Role in the DAKs**	**DAK Outputs**	**Basis for Inclusion**
1. Health interventions and associated recommendations: Overview of the health interventions and WHO recommendations included within this DAK. The list of health interventions is drawn from the universal health coverage menu of interventions compiled by WHO.	Contextualization: To understand the underlying guidance and interventions.	List of WHO Guidelines and related guidance for the DAK List of included health interventions	WHO Multisite Study
2. Generic personas: Depiction of the end users, supervisors, and related stakeholders who would be interacting with the digital system or involved in the clinical care pathway.	Contextualization: To understand the motivations and constraints of end users.	Description, competencies and essential interventions performed by targeted personas	CRDM
3. User scenarios: Narratives that describe how the different personas may interact with each other.	Contextualization: To understand how different personas interact and their potential engagement with the digital system.	Narrative of how the targeted personas may interact with each other during a workflow	CRDM
4. Generic business processes and workflows: Key processes and workflows for the identified health program area.	Contextualization and system design: To understand how activities are conducted within the health program and anchor the core data elements and decision-support logic.	Overview table presenting the key processes for the health program area Workflows for each of the processes, accompanied by annotations/notes	CRDM
5. Core data elements: Data elements required throughout the different points of the workflows and linked to terminology codes, such as ICD and other content standards.	System design and interoperability: To define the data elements required for clinical decision making or monitoring requirements, with terminology mappings to facilitate interoperability with other standards-based systems.	List of core data elements Annexed data dictionary with complete data specifications in spreadsheet format	WHO multisite study
6. Decision-support logic: Decision-support logic and algorithms in accordance with WHO guidelines.	System design and adherence to recommended clinical practice: To define the underlying algorithms and logic that need to be coded into the system.	Decision tables with inputs, outputs and triggers for each decision logic and scheduling logic for services, as a linked spreadsheet	WHO multisite study
7. Indicators and performance metrics: Core set of indicators that need to be aggregated for decision making, performance metrics, and sub-national and national reporting.	System design and programmatic monitoring: To define the calculations required for populating aggregate indicators for program management and health system monitoring derived from core data elements.	Indicators table with numerator and denominator derived from the data elements	WHO multisite study
8. Functional and nonfunctional requirements: Functions and capabilities the system must have to meet the end users’ (e.g., health workers’) needs (functional requirements).	System design: To know how the system should function to achieve the different business processes.	Illustrative list of functional and nonfunctional requirements linked to the different workflow activities and personas	CRDM

Abbreviations: CRDM, Collaborative Requirements Development Methodology; DAK, digital adaptation kit; ICD, International Classification of Diseases; WHO, World Health Organization.

This approach resulted in 4 initial DAKs: antenatal care,^36^ FP,^37^ sexually transmitted infections, and HIV. The DAKs also incorporated crosscutting considerations related to adolescent services, intimate partner violence, and self-care interventions. These first DAKs serve as the test cases for this approach within WHO and are now being introduced for country-level adaptation and further refinement. The Supplement provides examples of the different components of the DAKs across the various health areas. Additionally, the DAKs, including the spreadsheets detailing the data dictionary and decision-support algorithms are publicly available on the WHO website and hosted on a dedicated web portal.

The first 4 DAKs serve as the test cases for this approach within WHO and are now being introduced for country-level adaptation and further refinement.

### Learnings From Developing the First Set of DAKs

The development of these DAKs required several iterations and continuous discussions with a broad range of team members representing clinical, data (monitoring and evaluation, strategic information), and digital health domains. Among the different components, the development of the data dictionaries and decision-support logic was the most resource-intensive part of the process. This included the time to create the data dictionary and map the data elements to the appropriate terminology standards. In particular, common terms that did not have exact mappings to established terminologies, such as ICD, required a series of discussions with terminologists and clinicians to assess whether to compromise on “best fit” options or identify alternative ways of framing the data elements.

Applying the DAKs to SRH and HIV offered an opportunity to refine the methodology while also identifying practical implications for their use within this health program area. Some SRH domains, such as FP, have not consistently relied on routine health information systems for reporting and measuring coverage and quality of services. For example, tracking FP progress has often used indicators such as “modern contraceptive prevalence rate” and “unmet need” which rely on population-based surveys that cannot be calculated within routine health information systems.^38^^,^^39^ The introduction of digital systems that track service delivery in real-time as part of the routine health information systems required redefining the way indicators have traditionally been captured.^40^ In addition, it allows the opportunity to further analyze FP uptake at the clinic level by registering in real-time whether a woman has received or continued with her contraceptive method of choice, or whether she discontinued its use. While routine health information systems would provide more immediate data for programmatic decision making and health system monitoring, this shift in the availability of data generated would also need to be accompanied by training on effective data use.^39^

Another key consideration that shaped the content of the DAKs for SRH and HIV is that these are inherently sensitive topics, and digitizing content for these health program areas triggered additional privacy considerations. For example, in the case of FP, the team debated on the inclusion of marital status as a core data element for the generic version, as being unmarried or an adolescent may present barriers for accessing FP services in some contexts.^41^ In other instances, information on key populations, such as commercial sex workers or people who inject drugs, is very sensitive but also necessary for delivering person-centered care and ensuring the clients receive the appropriate recommended protocols. Although knowing this information is critical even when care is managed on paper-based systems, incorporating these data needs for a digital system where there are heightened concerns of data security and confidentiality presented questions on whether and how to record this information. Ultimately, the concerns of data security, privacy, confidentiality need to be addressed through national policies articulated within countries’ digital health strategies and other regulatory frameworks as a fundamental consideration for all individual-level health information.^42^

## DISCUSSION

This packaging of WHO guideline content into a standardized format for digital systems offers a novel approach to reinforce clinical, public health, and data recommendations, as well as a mechanism for driving interoperability and strengthening trust in the content of point-of-care systems. As such, DAKs serve as a foundational step in fulfilling WHO’s long-term vision of SMART guidelines for transforming guideline development, delivery, and application in the digital age.^12^ A key measure of success will be ensuring countries can adapt the generic DAKs according to their digital ecosystem and align them to their national health policies.^43^ This effort would require further understanding on promoting compliance of DAKs and ways to enhance usability among software developers and implementers. The next phase of testing and implementing DAKs at the country level will also be crucial to understanding how national policies deviate from global guidelines and to refining the information represented within the DAKs.

Considering that countries are at different stages of maturity in their adoption of digital systems, the DAKs may be applied across various scenarios. Countries that already have digital systems in place may use the information within the DAK to upgrade the underlying content and data to align with WHO recommendations and national guidelines. Other settings that have not yet digitized their systems can use the DAK to begin this process as a starting point for designing their digital system. Lastly, the DAKs promote data standards by directly incorporating codes from ICD and other established terminologies to reduce the risk of overlooking or incorrectly mapping to these standards.

Although this was initiated in SRH and HIV, efforts are underway for broadening the scope of health areas to offer a more comprehensive resource package. Additional health programs have recognized the value of this structured documentation, and this model is currently extended to client-facing self-care interventions and replicated for other domains, such as child health and immunization. As this process expands to new health domains, there will need to be a focus on identifying opportunities for integration and strengthening linkages across programmatic areas in ways that are also feasible for implementation practices.

Lastly, DAKs have the potential to add value to service delivery beyond digital systems and supplement derivative products for facilitating the adaptation and implementation of WHO guidelines at the country level.^44^ For example, the structured decision-support tables and algorithms can be applied to paper-based visual job aids for health workers. The streamlining of core data elements can also contribute to the standardization of data collection and indicator reporting, even if done on paper forms. As significant efforts were made within the DAKs to facilitate linkages between individual-level data to upstream indicators and monitoring needs, this content can help countries seeking to streamline information flow for targeted service delivery and harmonize paper-based health management information systems. Likewise, the workflows may also be used as instructional tools to help train health workers on the organization of care and coordination of service delivery across different actors.

DAKs have the potential to add value to service delivery beyond digital systems and supplement derivative products for facilitating the adaptation and implementation of WHO guidelines at the country level.

The WHO DAKs aim to catalyze a paradigm shift from static paper-based guidelines and data reporting forms to dynamic digital systems, to ensure the integrity of these tools and that countries can derive maximum value from their digital investments. As the field of digital health matures, efforts such as these that systematically promote adherence to evidence-based protocols and data use will be indispensable not only for improving health outcomes and health system performance but also for accelerating progress toward universal health coverage.

## Supplementary Material

GHSP-D-21-00320_Supplement.pdf

## Notes

**Peer Reviewed**

**First published online:** February 11, 2022.
