# Supplementary material for: Transitioning to Digital Systems: The Role of World Health Organization’s Digital Adaptation Kits in Operationalizing Recommendations and Interoperability Standards
Source: Glob Health Sci Pract. 2022 Feb 28;10(1):e2100320. doi: 10.9745/GHSP-D-21-00320 (PMC8885357; doi:10.9745/GHSP-D-21-00320)
Supplement: GHSP-D-21-00320_Supplement.pdf [file GHSP-D-21-00320_Supplement.pdf]

Supplement to: Tamrat T, Ratanaprayul N, Barreix *M*, et al. Transitioning to digital systems: the role of World Health Organization digital adaptation kits in operationalizing recommendations and standards. *Glob Health Sci Pract*. 2022;10(1):2100320. https://doi.org/10.9745/GHSP-D-21-00320

Supplement. Examples of different components of World Health Organization Digital Adaptation Kit

Supplement Figure 1. Example of personas (component 2) in family planning Digital Adaptation Kit

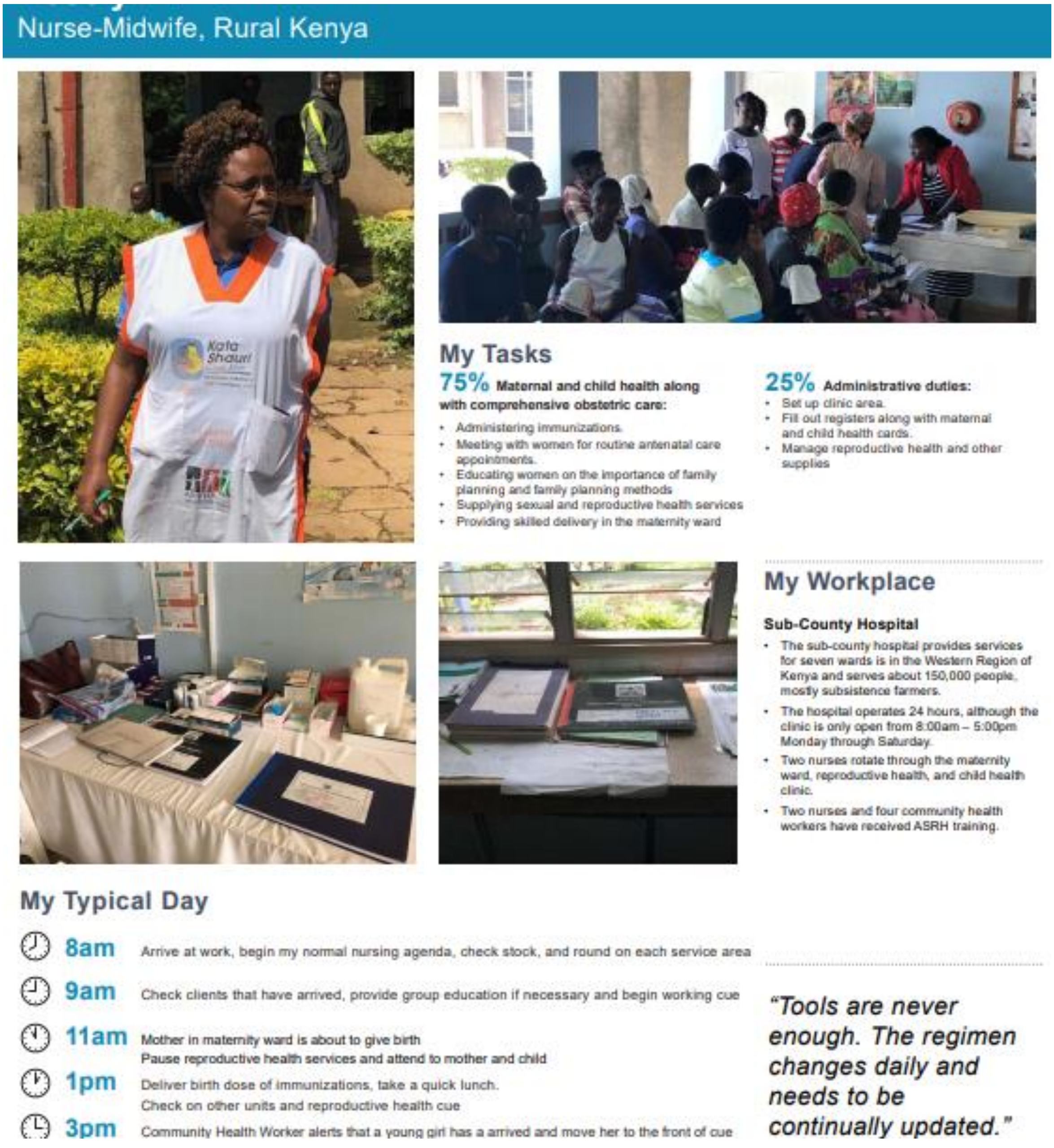

Supplement to: Tamrat T, Ratanaprayul N, Barreix *M*, et al. Transitioning to digital systems: the role of World Health Organization digital adaptation kits in operationalizing recommendations and standards. *Glob Health Sci Pract*. 2022;10(1):2100320. https://doi.org/10.9745/GHSP-D-21-00320

Supplement Figure 2. Example of workflow (component 4) in Sexually Transmitted Infection Digital Adaptation Kit

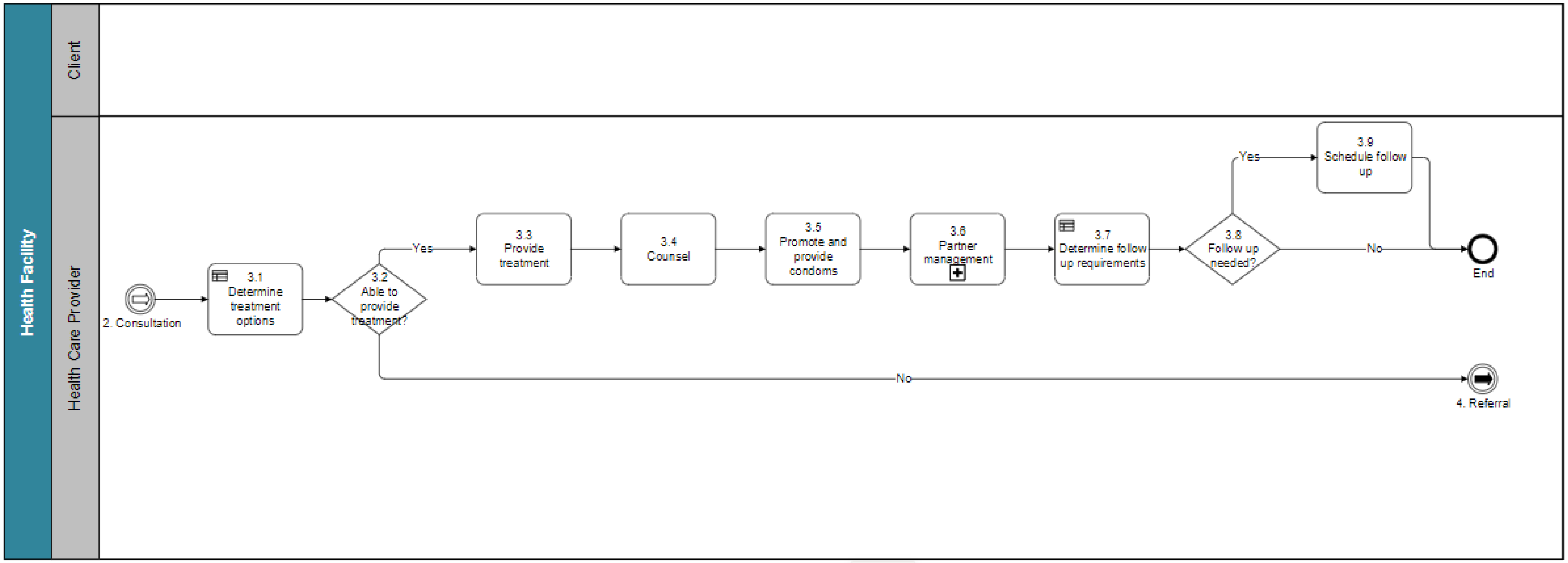

Supplement to: Tamrat T, Ratanaprayul N, Barreix *M*, et al. Transitioning to digital systems: the role of World Health Organization digital adaptation kits in operationalizing recommendations and standards. *Glob Health Sci Pract*. 2022;10(1):2100320. https://doi.org/10.9745/GHSP-D-21-00320

Supplement Figure 3. Example of data dictionary (component 5) in HIV Digital Adaptation Kit

Activity ID	Data Element Label	Description and Definition	Multiple Choice Type (if applicable)	Data Type	Input Options	ICD-11 Code	ICD-11 URI	ICD-11 Comments / Considerations
**HIV.H. Record outreach and result**	**Client outcome**	The outcome for the client which is used for reporting retention. Included in total attrition from antiretroviral therapy (ART): death, stopped treatment and lost to follow-up. On antiretroviral therapy (ART) is included so that client outcome	**Select one**	**Coding**	**N/A**			
	Lost to follow up	Twenty-eight days or more since last missed appointment	Input Option	Codes	Lost to follow-up	Not classifiable in ICD-11		
	Transferred out	The client transferred to another facility	Input option	Codes	Transferred out	Not classifiable in ICD-11		
	Death (documented)	People living with HIV previously on antiretroviral therapy (ART) who are confirmed to have died from any cause.	Input option	Codes	Death (documented)	MH13	http://id.who.int/icd/entity/1876291018	Code title: Unattended death
	Refused (stopped) treatment	Client was contacted and confirmed to have stopped antiretroviral therapy (ART). (Reasons may include stigma and discrimination, faith healing, etc.)	Input option	Codes	Refused (stopped) treatment	QC49	http://id.who.int/icd/entity/1626712964	Code title: Personal history of noncompliance wit[illegible]
	On ART	Client is currently taking antiretroviral medication	Input option	Codes	On ART	XM63K0	http://id.who.int/icd/entity/1722279370	Code title: Anti-infe[illegible] antiviral
	Date client outcome changed	The date on which the client's outcome (lost to follow up, transferred out, death (documented), or refused (stopped) treatment changed	N/A	Date	N/A			
	Transfer confirmed	Select if transfer to another facilitiy is confirmed	N/A	Boolean	True/False	Not classifiable in ICD-11		True False
	Transfer To facility	Name of health facility client was transferred to	Select one	Coding	N/A	Not classifiable in ICD-11		Free text field
	Date of transfer out	The date the client transferred out of the facility to be provided with care at another facility	N/A	Date	N/A	Not classifiable in ICD-11		Date
	Adherence assessment	Whether client is adherent or not to antiretroviral therapy regimen per national guidelines (immunological or virological monitoring)		Boolean	True/False			Not classifiable in I[illegible] 11

Supplement to: Tamrat T, Ratanaprayul N, Barreix *M*, et al. Transitioning to digital systems: the role of World Health Organization digital adaptation kits in operationalizing recommendations and standards. *Glob Health Sci Pract*. 2022;10(1):2100320. https://doi.org/10.9745/GHSP-D-21-00320

Figure 4. Example of decision-support logic table (component 6) in ANC DAK

Decision ID	ANC.DT.26 Pre-eclampsia risk counselling								
Business Rule	If the woman has risk factors for developing pre-eclampsia, provide counselling to reduce risk of developing pre-eclampsia								
Trigger	ANC.B6. Collect woman's profile & history								
Input(s)			Output	Action			Annotations	Reference(s)
'Number of fetuses' ≥ 2	'Gestational age' ≥ 12 weeks	'Population with low dietary calcium intake' =	'Risk of pre-eclampsia' = TRUE	Provide pre-eclampsia risk counselling	'Amount of daily aspirin prescribed until delivery' = 75 mg	1.5 g ≤ 'Amount of calcium supplements provided' ≤ 2 g	The woman is at risk of developing pre-eclampsia: provide counselling; prescribe aspirin 75 mg daily until delivery (starting at 12 weeks gestation) and ensure that she continues to take her daily calcium supplement of 1.5–2 g until delivery for pre-eclampsia risk	Pregnancy, childbirth, postpartum and new care guide (2015): C3 *(1)* WHO pre-eclampsia and eclampsia
'Past pregnancy complications' = 'Pre-eclampsia'	'Gestational age' ≥ 12 weeks	'Population with low dietary calcium intake' =	'Risk of pre-eclampsia' = TRUE	Provide pre-eclampsia risk counselling	'Amount of daily aspirin prescribed until delivery' = 75 mg	1.5 g ≤ 'Amount of calcium supplements provided' ≤ 2 g		Pregnancy, childbirth, postpartum and new care guide (2015): C3 *(1)* WHO pre-eclampsia and eclampsia
'Past pregnancy complications' = 'Eclampsia'	'Gestational age' ≥ 12 weeks	'Population with low dietary calcium intake' =	'Risk of pre-eclampsia' = TRUE	Provide pre-eclampsia risk counselling	'Amount of daily aspirin prescribed until delivery' = 75 mg	1.5 g ≤ 'Amount of calcium supplements provided' ≤ 2 g		Pregnancy, childbirth, postpartum and new care guide (2015): C3 *(1)* WHO pre-eclampsia and eclampsia
'Past pregnancy complications' = 'Convulsions'	'Gestational age' ≥ 12 weeks	'Population with low dietary calcium intake' =	'Risk of pre-eclampsia' = TRUE	Provide pre-eclampsia risk counselling	'Amount of daily aspirin prescribed until delivery' = 75 mg	1.5 g ≤ 'Amount of calcium supplements provided' ≤ 2 g		Pregnancy, childbirth, postpartum and new care guide (2015): C3 *(1)* WHO pre-eclampsia and eclampsia
'Existing chronic health conditions' =	'Gestational age' ≥ 12 weeks	'Population with low dietary calcium intake' =	'Risk of pre-eclampsia' = TRUE	Provide pre-eclampsia risk counselling	'Amount of daily aspirin prescribed until delivery' = 75 mg	1.5 g ≤ 'Amount of calcium supplements provided' ≤ 2 g		Pregnancy, childbirth, postpartum and new care guide (2015): C3 *(1)* WHO pre-eclampsia and eclampsia
'Existing chronic health conditions' =	'Gestational age' ≥ 12 weeks	'Population with low dietary calcium intake' =	'Risk of pre-eclampsia' = TRUE	Provide pre-eclampsia risk counselling	'Amount of daily aspirin prescribed until delivery' = 75 mg	1.5 g ≤ 'Amount of calcium supplements provided' ≤ 2 g		Pregnancy, childbirth, postpartum and new care guide (2015): C3 *(1)* WHO pre-eclampsia and eclampsia
'Existing chronic health conditions' =	'Gestational age' ≥ 12 weeks	'Population with low dietary calcium intake' =	'Risk of pre-eclampsia' = TRUE	Provide pre-eclampsia risk counselling	'Amount of daily aspirin prescribed until delivery' = 75 mg	1.5 g ≤ 'Amount of calcium supplements provided' ≤ 2 g		Pregnancy, childbirth, postpartum and new care guide (2015): C3 *(1)* WHO pre-eclampsia and eclampsia
'Existing chronic health conditions' =	'Gestational age' ≥ 12 weeks	'Population with low dietary calcium intake' =	'Risk of pre-eclampsia' = TRUE	Provide pre-eclampsia risk counselling	'Amount of daily aspirin prescribed until delivery' = 75 mg	1.5 g ≤ 'Amount of calcium supplements provided' ≤ 2 g		Pregnancy, childbirth, postpartum and new care guide (2015): C3 *(1)* WHO pre-eclampsia and eclampsia
